# Dysembryoplastic Neuroepithelial Tumor: A Case Report of A Benign Intracranial Lesion Masquerading as Seizure Disorder

**DOI:** 10.7759/cureus.64047

**Published:** 2024-07-07

**Authors:** Garima S Agarwal, Anil K Agrawal, Daksh Singhal, Jayashree Bhawani

**Affiliations:** 1 Pathology, Jawaharlal Nehru Medical College, Datta Meghe Institute of Higher Education & Research, Wardha, IND; 2 Psychiatry, Rajarajeshwari Medical College, Bengaluru, IND

**Keywords:** temporal lobe seizure, s-100, pilocytic astrocytomas, oligodendrocytes, cerebral cortex, floating neurons, glial nodules, glial fibrillary acidic protein, seizure, dysembryoplastic neuroepithelial tumour (dnt)

## Abstract

The uncommon, benign dysembryoplastic neuroepithelial tumor (DNET, WHO grade 1) is frequently linked to epilepsy. It is a glioneuronal neoplasm in the cerebral cortex of children or young adults defined by the presence of a pathognomonic glioneuronal element that may be linked to glial nodules and activating mutations of fibroblast growth factor receptor 1 (*FGFR1*) (CNS WHO grade 1 according to WHO classification of CNS and pituitary tumors, 2021 ). The cerebral cortex is primarily affected. The most frequent areas are the temporal lobe, particularly the medial lobe, frontal lobe, and other cortex. This study reports the instance of a 31-year-old male who had a history of seizures for the past 20 years and complained of a sudden headache and vomiting at the hospital. MRI revealed a cortical-based lesion in the left posterior temporo-occipital region. A biopsy sample was sent for histopathological examination. DNETs are usually benign, non-recurring lesions and rarely can be a malignant transformation. Although they are frequently stable tumors, surgical excision seldom results in recurrence.

## Introduction

Benign mixed glioneuronal neoplasms known as dysembryoplastic neuroepithelial tumors (DNETs) are common in children and young adults aged 10-14 years old, being identified as having the highest peak incidence [1]. Males are more commonly affected than females [2]. The incidence of the disease is about 0.2% in patients over 20 years. They were initially reported by Damas-Duport in 1998, and their histopathological features include the presence of cells that resemble oligodendrocytes [3]. DNETs may exhibit partial complex seizures, which are the most prevalent type and are occasionally accompanied by papilledema and headaches. They may also exhibit chronic drug-resistance seizures [4]. This study presents a case of a 31-year-old male with a history of seizures who presented to the hospital with a complaint of sudden headache and vomiting. Following imaging scans that demonstrated a well-defined mass, a biopsy was performed, and the histopathological examination results pointed to the diagnosis of DNET. This article aims to create awareness amongst health professionals who may encounter similar cases.

## Case presentation

A 31-year-old male with a history of seizures since 10 years of age, presented to the hospital with a complaint of sudden headache and vomiting. The patient's medical history was notable for the onset of seizures at the age of 10 years, which were well-controlled with antiepileptic medications, i.e., tablet levetiracetam 500 mg BD.

The patient was orientated to time, place, and person upon admission. He had an oxygen saturation (SpO2) of 92% and a blood pressure of 116/82 mmHg [5]. The remaining critical indicators were all within normal ranges. The neurological assessment revealed no focal deficits. However, the sudden and severe headache accompanied by vomiting raised concerns and prompted further investigations. There was no history of ENT bleed and no history of bladder/bowel complaints.

**Figure 1:**
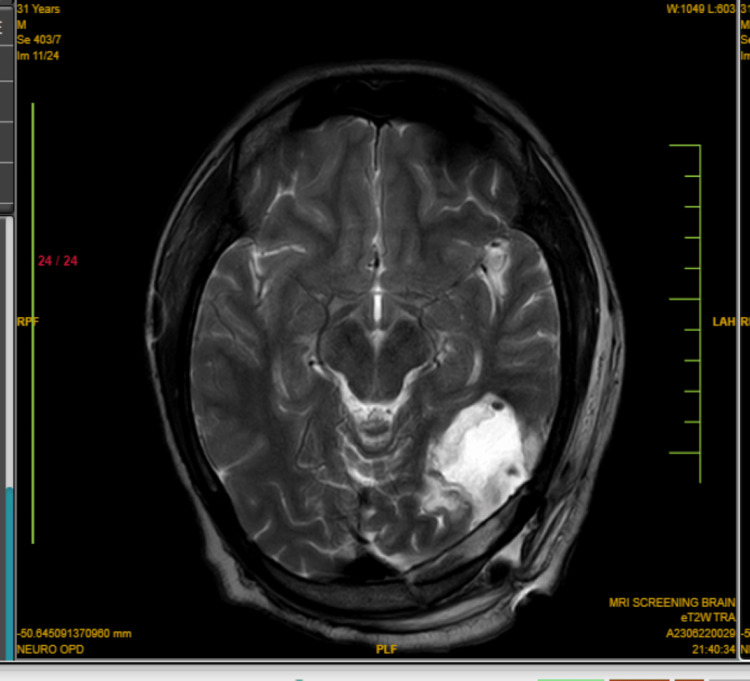
MRI brain shows multiple areas of postoperative calvarial defect in the left temporo-occipital region with heterogeneously T2/FLAIR hyperintense collection noted in the subdural space. FLAIR: Fluid-attenuated inversion recovery

MRI revealed a cortical-based lesion in the left posterior temporo-occipital region. The lesion exhibited T1 hypointensity, T2 hyperintensity, and fluid-attenuated inversion recovery (FLAIR) heterogeneously hyperintense. The patient underwent a left occipital craniotomy for the excision of the lesion. A 3 x 3 cm craniotomy was done (Figure 1).

**Figure 2:**
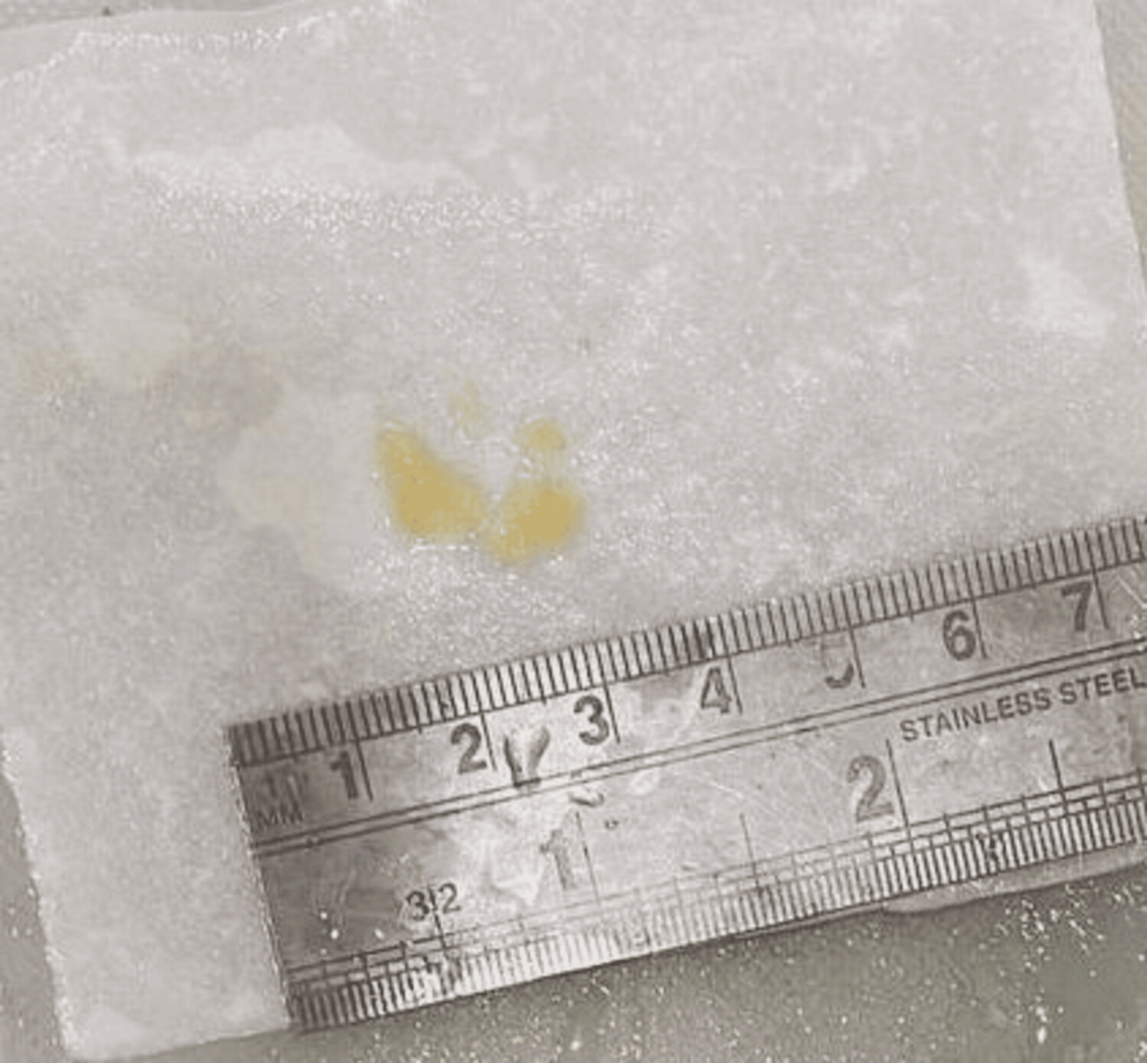
Multiple, irregular, greyish-white tissue bits aggregating 0.5 x 0.5 cm

A biopsy from the excised brain specimen was sent for histopathological examination. Grossly, the tissue bits were multiple, irregular, greyish-white aggregating 0.5 x 0.5 cm (Figure 2). The entire tumor was excised and post-operatively the patient was shifted to neuro ICU where he was managed with antibiotics such as an injection of ceftriaxone 1 g IV BD and injection of amikacin OD, antacids i.e., injection of pantoprazole 40 mg OD, antiepileptics, and other supportive measures. The post-operative course was unremarkable and the patient was neurologically stable and shifted to the ward. Adjuvant treatment was not required as the entity was benign. Follow-up was done after 15 days and the patient did not exhibit any symptoms.

**Figure 3:**
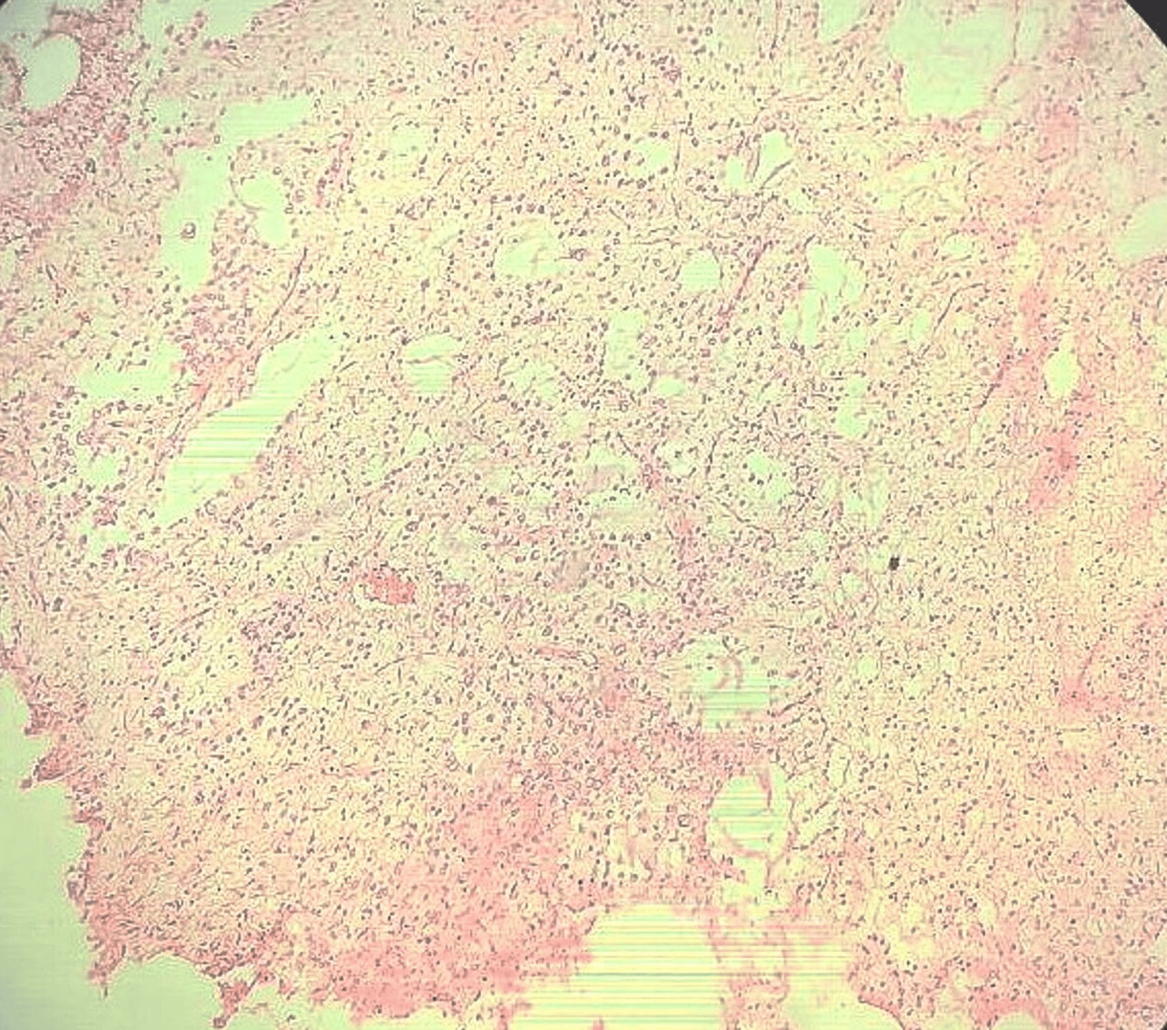
Low-power view image (10x). This microscopic image shows oligodendrocyte-like cells arranged along bundles of axons separated by a myxoid matrix.

**Figure 4:**
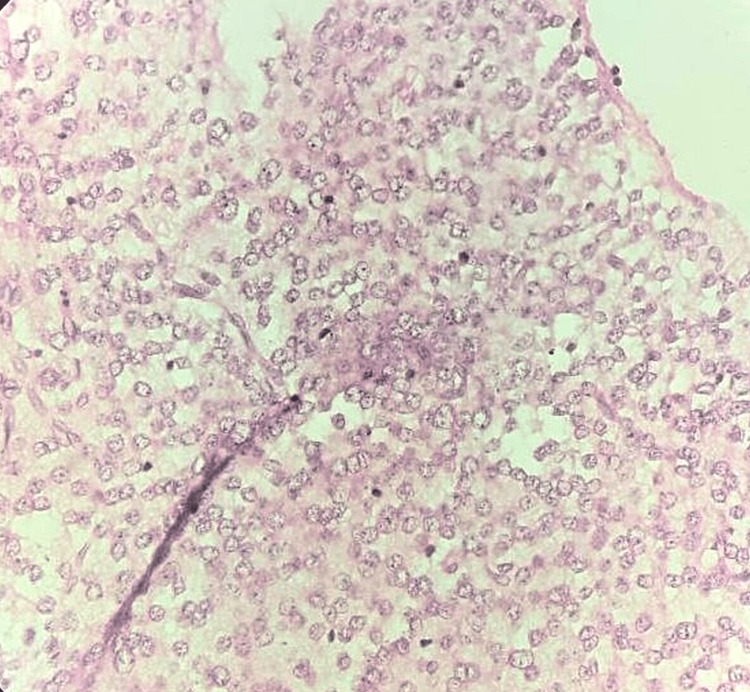
High-power view image (40x). This microscopic image shows multiple oligodendrocyte-like cells separated by a myxoid matrix containing pathognomonic floating neurons.

Microscopically, a section from the biopsy showed oligodendrocyte-like cells arranged along bundles of axons separated by a myxoid matrix that contains floating neurons. There was no evidence of necrosis or mitotic figures. All histopathological features suggested the diagnosis of DNET (WHO grade 1). No immunohistochemical (IHC) markers were performed (Figure 3 and Figure 4).

## Discussion

DNETs are rare neuroepithelial tumors described by Daumas-Duport in 1988 [3]. DNETs are low-grade, mixed neuronal-glial neoplasms. WHO (2021) categorized them as grade 1 central nervous system tumors [6]. There have been documented cases frequently linked to mutations in the germline *FGFR1 *p.R661P [5].

The temporal lobe is the most common site for these benign, supra-tentorial tumors. A history of pharmacologically intractable seizures is a common feature among the majority of patients under 20 years of age [6]. Twenty percent of surgically excised tumors from patients whose epilepsy was not treatable with medicine have been reported to have these neoplasms [4]. Partial complex seizures are the most prevalent type of seizure semiology, then generalized tonic-clonic and simple partial seizures came next [4].

Histologically, DNETs can be divided into three categories: non-specific, complex, and simple. Although they are frequently stable tumors, surgical excision seldom results in their recurrence [6]. The tumors are primarily seen in the mesial temporal lobe. However, they can also be found in the frontal and parieto-occipital lobes. They commonly range in size from 10-25 mm [4]. DNET might be a well-defined, solitary nodular, or weakly delineated mass lesion, depending on its morphology [1]. Magnetic resonance imaging (MRI) and computed tomography (CT) commonly show cortical cystic or multicystic lesions [5]. Calcifications and cystic alterations are frequently noticed in DNET [5]. Histology is the abundance of mucinous matrix, floating neurons, and small round cells known as oligodendroglia-like cells (OLCs) that lack dysplasia [1].

Other glioneuronal or glial tumors, such as pleomorphic xanthoastrocytomas, gangliogliomas, oligodendrogliomas, and pilocytic astrocytomas, are included in the differential diagnosis. DNET is a challenging diagnosis because no pathognomic findings exist [4]. While the floating neurons express neuronal markers such as synaptophysin neurofilament, NeuN, neuron-specific enolase, microtubule-associated protein 2 (*MAP2*), and class-III beta-tubulin, the bulk of OLCs are significantly positive for S100 protein and Oligodendrocyte transcription factor (*OLIG2*) [4]. The benign nature of DNET shows a low proliferative index Ki-67 [7]. IHC markers are useful in confirming the diagnosis but in this case, no immunohistochemical markers were performed.

In this case, radiological imaging suggested that the lesion was neoplastic. However, the biopsy result following tumor excision revealed no evidence of a high-grade lesion. For these patients, monitoring is crucial to identify any recurrences.

## Conclusions

This case study discusses DNETs that can develop into malignancies, have *FGFR1 *mutations, and induce symptomatic intracranial hemorrhage. When tumors arise outside the temporal lobe and exhibit enhancement on post-contrast-enhanced T1 weighted imaging, malignant transformation of DNETs should be considered a differential diagnosis in patients suspected of DNETs. IHC markers can help in further diagnosis. The positive stains are S100, OLIG2, platelet-derived growth factor receptor alpha (*PDGFRA*), NeuN, and alcian blue. Generally, no adjuvant therapy is required. Complete surgical resection is still the gold standard of treatment because it is linked to 80-100% seizure-free results in most instances.
